# Impact assessment of the incorporation of the rotavirus vaccine in the province of San Luis – Argentina

**DOI:** 10.1017/S0950268819001936

**Published:** 2019-11-27

**Authors:** S. García Martí, F. Augustovski, L. Gibbons, V. Loggia, A. Lepetic, J.A. Gómez, A. Pichón Riviere

**Affiliations:** 1 Instituto de Efectividad Clínica y Sanitaria, Dr Emilio Ravignani 2024 (C1414CPV), Buenos Aires, Argentina; 2 GSK, Av. Carlos Casares 3690, B1644, Victoria, Buenos Aires, Argentina

**Keywords:** Acute gastroenteritis, interrupted time-series, rotavirus gastroenteritis, rotavirus vaccination, vaccination impact

## Abstract

Rotavirus (RV) is the main cause of acute gastroenteritis (AGE) in young children. The San Luis province of Argentina introduced RV vaccination in May 2013. We estimate vaccine impact (RVI) using real-world data. Data on all-cause AGE cases and AGE-related hospitalisations for San Luis and the adjacent Mendoza province (control group) were obtained and analysed by interrupted time-series methods. Regardless of the model used for counterfactual predictions, we estimated a reduction in the number of all-cause AGE cases of 20–25% and a reduction in AGE-related hospitalisations of 55–60%. The vaccine impact was similar for each age group considered (<1 year, <2 years and <5 years). RV vaccination was estimated to have reduced direct medical costs in the province by about 4.5 million pesos from May 2013 to December 2014. Similar to previous studies, we found a higher impact of RV vaccination in preventing severe all-cause AGE cases requiring hospitalisation than in preventing all-cases AGE cases presenting for medical care. An assessment of the economic value of RV vaccination could take other benefits into account in addition to the avoided medical costs and the costs of vaccination.

## Introduction

Rotavirus (RV) infection is the main cause of severe diarrhea and acute gastroenteritis (AGE) in infants and young children, while diarrhea is the second most frequent cause of childhood death [1]. Across the world, virtually all children will have been infected with RV before the age of 5 years and suffered an RV gastroenteritis (RVGE) with diarrhea, vomiting and other symptoms like nausea and stomach cramps. RV is transmitted primarily via the faecal-oral route and transmission occurs either directly between persons or via touching contaminated surfaces, where the virus may remain infectious for extended periods [2, 3].

Effective, live-attenuated RV vaccines have been available and licensed since 2006; in 2009, the WHO recommended implementation of RV vaccination in the routine childhood vaccination programs in all countries, and in priority in countries with high AGE-related childhood mortality [4].

Most Latin American countries were among the early adopters of this policy recommended by the WHO. Some countries in the continent, Chile, Uruguay and Argentina among them, with very low-diarrhea-associated childhood mortality rates did not at first adopt RV vaccination widely. However, these countries allowed primary-care physicians to administer RV vaccination on an individual basis [5]. Contrary to Chile and Uruguay where population-wide RV vaccination has not yet been introduced, Argentina included this in its national routine childhood vaccination program in January 2015 [6]. Like all the vaccines in the program, RV vaccination is provided at zero out-of-pocket costs for all children.

The clinical care and possible treatment of AGE cases do not depend on the specific causative agent so the common practice is that laboratory testing of biological specimens is relatively uncommon, particularly in cases presented in primary care. Studies in Argentina found that 16.8% of AGE cases treated in outpatient settings [7] and 42% of hospitalised AGE cases were RVGE [8].

A single Argentinian province, San Luis, included RV vaccination in its routine childhood vaccination program in May 2013, almost 2 years earlier than the national one. This divergence in the time of introduction presents an excellent and relatively rare opportunity for using a quasi-experimental design to evaluate the early impact of RV vaccination in ordinary clinical practice.

We performed an impact study at the population level aiming at measuring the early impact of RV vaccination by using health outcomes data pre- *vs.* post-introduction of the RV vaccine. The impact of RV vaccination is estimated by comparing the observed health outcomes in San Luis province post RV vaccine introduction with a counterfactual prediction of what the outcomes would have been without the vaccine.

## Methods and material

The study period extended from 1 January 2008 to 31 December 2016. The RV vaccination was introduced in San Luis in May 2013, so the pre-vaccination period ended on 30 April 2013, at which date the post-vaccination period began. No transition period was considered. A full 2-dose vaccination schedule must be completed before the infant is 24 weeks old.

AGE is a mandatory notifiable disease in Argentina, which is to be reported to the National Health Surveillance System (SNVS). A clinical module of SNVS is used to collect information from all ‘medical consultations’ regardless of the setting in which they take place (primary care, ambulatory services, emergency rooms and hospital units). Another module collects information from laboratory surveillance based on the biological specimens received and analysed by SNVS laboratory networks. The clinical module SNVS data was used to estimate the incidence of all-cause AGE at the provincial level in children aged <5 years.

The data on all-cause AGE-associated hospitalisations were derived from hospital discharge (HD) data from the Public Health Sector at the provincial level. Admissions with the discharge diagnoses ‘intestinal infection due to a virus and other specified organisms’ or ‘diarrhea and gastroenteritis of presumed infectious origin’ (CIE-10) were considered as AGE-associated and included in the study. Only one AGE-related death occurred during the study period, so this outcome was not analysed.

No major changes in the methods for registering AGE cases, hospitalisations or in the health care system of any of the two provinces were identified as occurring during the study period. It was therefore assumed that the difference between the observed and predicted incidence of AGE and the number of AGE-associated hospitalisations could be attributed to RV vaccination. The robustness of this assumption was assessed by repeating the statistical analyses with randomly selected five hypothetical time points for the RV vaccine introduction. The assumption would be considered robust if these analyses with hypothetical time points for the intervention showed no effect in any of them.

Only when the actual date of vaccine introduction was considered in the model could we identify an effect in terms of reductions in AGE cases and hospitalisations in the analysis.

An estimate of the direct healthcare costs avoided due to the impact of RV vaccination was derived based on unit cost estimates for outpatient care and hospitalisations from an economic evaluation of RV vaccine published in 2011 [9]. The cost estimates were updated to 2014 values by using inflation data from the general Provincial Bureau of Statistics and Census in San Luis.

After the introduction of the RV vaccination in May 2013, a coverage rate for the 2-dose schedule of 99% was achieved already in 2014 and more or less maintained throughout the study period, so full coverage was assumed.

### Statistical analyses

The data were analysed by interrupted time-series methods with the aim of predicting what the outcomes would have been in San Luis, if the RV vaccination had not been introduced. The specific model used was an indirect, counterfactual Bayesian prediction [10], a method that generalises the widely used difference-in-differences approach to time-series analyses by explicitly modelling the counterfactual of a time series observed both before and after an intervention. It improves on existing methods in two aspects: it provides a fully Bayesian time-series estimate for the effect; and it uses model averaging to construct the most appropriate synthetic control for modelling the counterfactual. This powerful approach to constructing the counterfactual is based on the idea of combining a set of candidate predictor variables into a single ‘synthetic control’ [11, 12]. There are three sources of information available for constructing an adequate synthetic control. The first is the time-series behaviour of the outcome prior to the intervention. The second is the behaviour of other time series that were predictive of the outcome series prior to the intervention. In a Bayesian framework, a third source of information for inferring the counterfactual is the available prior knowledge about the model parameters, for example as elicited by previous studies.

We combine these sources of information using a state-space time-series model, where one component of state is a linear regression on the contemporaneous predictors. The framework of our model allows us to choose from among a large set of potential controls by placing a spike-and-slab prior to the set of regression coefficients and by allowing the model to average over the set of controls [13]. We then compute the posterior distribution of the counterfactual time series given the value of the outcome series in the pre-intervention period, along with the values of the controls in the post-intervention period. Subtracting the predicted from the observed outcome during the post-intervention period gives a semiparametric Bayesian posterior distribution for the causal effect.

San Luis data from January 2008 to the end of April 2013 were used to model the counterfactual prediction of the number of AGE cases and related hospitalisations that would have occurred from May 2013 until the end of 2016 regarding AGE cases and until the end of 2015 regarding hospitalisations if the RV vaccination had not been introduced.

In addition, data from the Mendoza province, which did not introduce RV vaccination until January 2015, were used as a control and San Luis AGE events were adjusted using these data. The Mendoza province is adjacent to San Luis with similar weather, geography and sanitation conditions and prior to the introduction of RV vaccination in San Luis, the incidences of all AGE cases and AGE-associated hospitalisations were equivalent in both provinces. As RV vaccination was introduced in Mendoza in January 2015, concurrently with its introduction in the National Immunisation Program, this model with Mendoza data as a control group was used to estimate the impact of RV vaccination in San Luis until the end of 2014. This prediction model assumes that the relationship between the outcomes observed in San Luis and Mendoza prior to the start of RV vaccination in May 2013 would have remained stable in the prediction period, if vaccination had not been introduced.

The predicted number of AGE cases and AGE-related hospitalisations, the average number of cases and hospitalisations averted per time period, the relative effect of RV vaccination and the cumulative number of cases and hospitalisations averted were estimated with 95% confidence intervals (CI). Time-trend analyses were illustrated in combined diagrams showing observed and predicted outcomes over time, point-wise impact of the RV vaccine and the cumulative impact over the time period May 2013 to December 2014. Results are presented for 3 age groups: i.e. children younger than 1 year, 2 years and 5 years.

The analyses were performed by means of the R statistical package with an addition specifically designed for this type of analysis [10].

### Ethics

The data collected and analysed were anonymised (without personal identifiers) and no information for individual persons was evaluated, as only provincial level health statistical data were used. Individual informed consent is not required for this type of study, but the provincial health authorities were consulted about the study procedures and agreed with them.

## Results

### Incidence of all-cause AGE

**Fig. 1.**
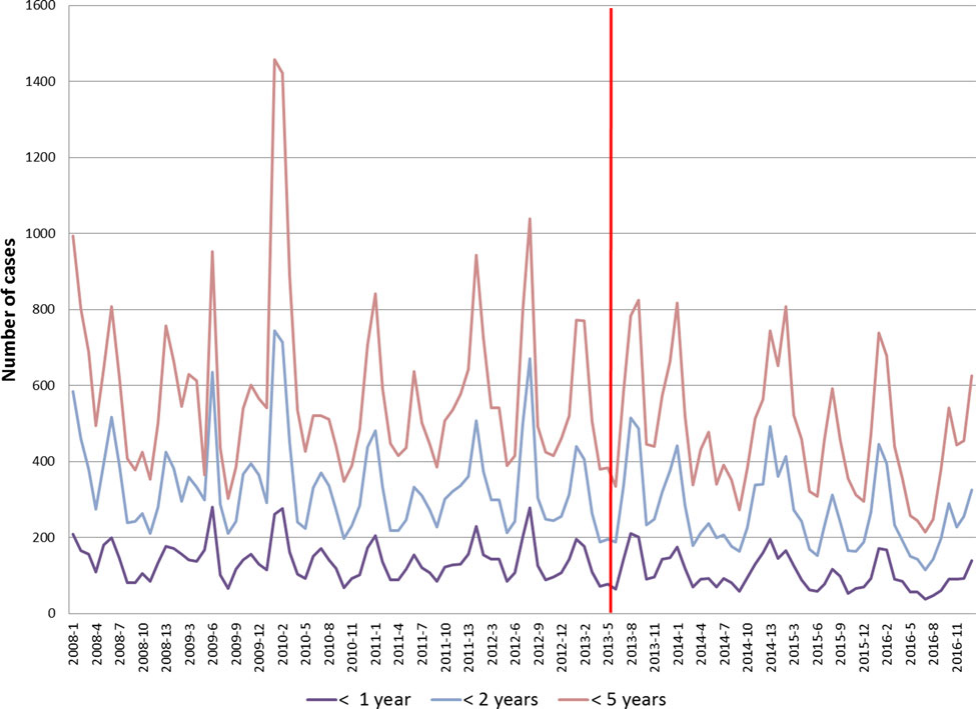
Number of AGE cases for different age groups per 4-week periods for San Luis. AGE, acute gastroenteritis.

Figure 1 presents the number of all-cause AGE cases per 4-week period in San Luis over the entire study period. A similar figure for Mendoza may be found in the Supplementary Material available online for this article (Fig. SM1). In each figure, the vertical red line marks the respective time points of the start of RV vaccination.

**Fig. 2.**
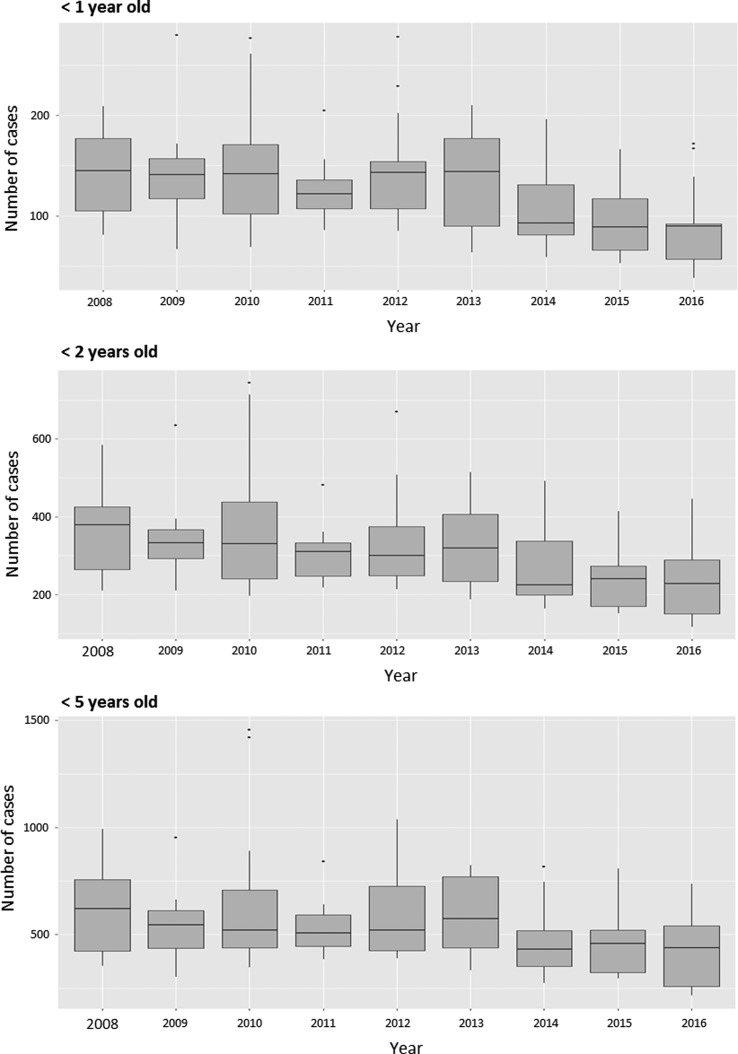
Boxplot of the distribution of the number of AGE cases per 4-week period each year of the study period in children in different age groups in San Luis. AGE, acute gastroenteritis.

The boxplot diagram in Figure 2 shows the distribution of the number of AGE cases per 4-week period in children younger than 5 years old in San Luis for each year, stratified into the age groups <1 year, <2 years and <5 years. No trends were discernible over the years before 2013, but for 2014 and onwards the medians were below the medians observed before 2013.

#### Time-trend analysis for AGE cases in San Luis (2008–2016)

The results summarised in the upper part of Table 1 indicate a statistically significant relative reduction of 19% (95% CI 6%–33%) of all-AGE cases in children younger than 5 years with slightly higher relative reductions in the two youngest age groups. The cumulative number of cases averted in children aged less than 1, 2 and 5 years until the end of 2016 were 1636 (95% CI 684–2587), 3698 (95% CI 1489–5895) and 5381 (95% CI 1573–9183), respectively (Figs SM2 and 3). Regarding the cumulative number of cases averted and the relative reduction in the 1 < 2 and 2 < 5 groups were 2063 (95% CI 786–3469) cases averted and 21% reduction (95% CI 8%–36%) for the 1 < 2 and 1689 (95% CI 19–3473) cases averted and 14% reduction (95% CI 0%–29%) respectively.

**Table 1.** Time-trend analysis of AGE cases per 4-week period in San Luis with and without adjustment using Mendoza data as control

	Average number of cases pre-vaccination	Average number of cases post-vaccination	Predicted number of cases without vaccination (95% CI)	Absolute effect averted # of cases (95% CI)	Relative effect (95% CI)	*P* value
Age group						
Model 1: Time-trend analysis for San Luis (2008–2016)						
<1 year	141	105	139 (119–158)	−34 (−54 to −14)	−25% (−39% to −10%)	0.001
<2 years	343	264	342 (295–387)	−77 (−123 to −31)	−23% (−36% to −9%)	0.001
<5 years	591	475	587 (508–667)	−112 (−191 to −33)	−19% (−33% to −6%)	0.003
Model 2: Time-trend analysis for San Luis adjusted by Mendoza as control (2008–2014)						
<1 year	141	119	145 (122–167)	−27 (−4 to −49)	−18% (−3% to −34%)	0.01
<2 years	343	291	360 (305–413)	−69 (−14 to −123)	−19% (−4% to −34%)	0.010
<5 years	591	508	636 (540–735)	−127 (−32 to −227)	−20% (−5% to −36%)	0.006

#: Number; CI: confidence interval; AGE: acute gastroenteritis.

#### Time-trend analysis of AGE cases in San Luis adjusting with Mendoza data as control (2008–2014)

These results summarised in the lower part of Table 1 show a statistically significant relative reduction of 20% (95% CI 5%–36%) of all-cause AGE cases in children younger than 5 years with similar relative reductions for the two youngest groups. Regarding the cumulative number of cases averted and the relative reduction in the 1 < 2 and 2 < 5 groups were 927 (95% CI 185–1659) cases averted and 20% reduction (95% CI 4%–35%) for the 1 < 2 and 1486 (95% CI 466–2454) cases averted and 24% reduction (95% CI 7%–39%) respectively.

**Fig. 3.**
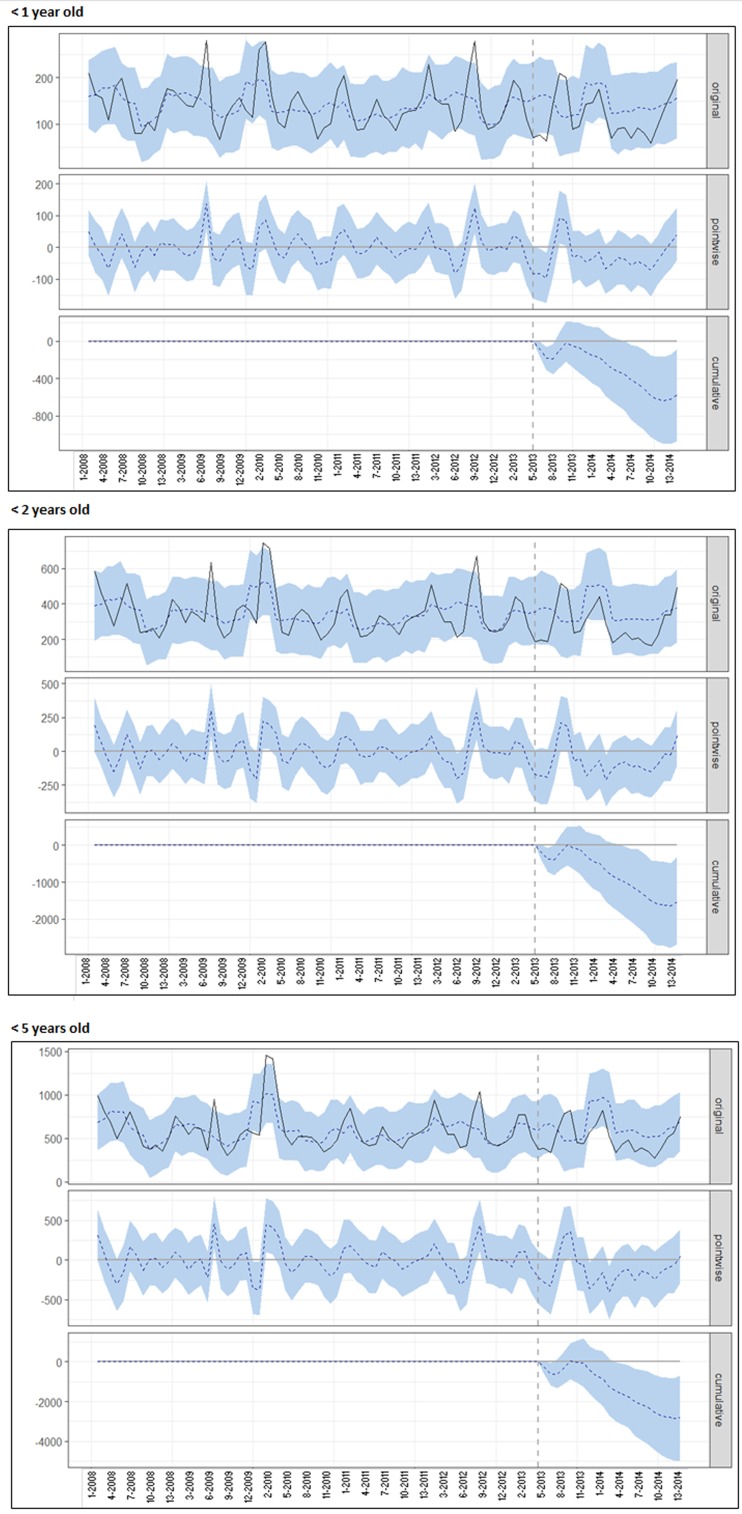
Time-trend analysis for AGE cases in different age groups in San Luis, after adjusting with Mendoza data as control group. This figure shows the number of cases or HDs for the different age groups by epidemiological week (starting in January 2008). The upper graph (original) contains the observed values (full line) and the model predicted values in the case of no intervention (dotted line). The middle (pointwise) graph shows the difference between the observed and predicted value by period under analysis and the lower (cumulative) graph shows the cumulative difference throughout the period under analysis. The vertical dotted line represents the time of introduction of the vaccine (May 2013). The areas shadowed in light blue refer to the 95% CI of the estimate in the upper graph (original), of the difference for each time point (pointwise) in the middle graph and of the cumulative difference in the lower graph (cumulative). AGE: acute gastroenteritis.

Figure 3 illustrates the time-trend analysis of all-cause AGE cases for San Luis (from January 2008 until the end of 2014) using Mendoza data as control. For each age group, the top panel shows the observed (full line) and predicted (dotted line) number of AGE cases; the middle panel shows the time-point difference between observed and predicted AGE cases and the bottom panel shows the cumulative difference between the observed and predicted AGE cases after the start of RV vaccination until the end of 2014. The cumulative number of cases averted in children aged less than 1, 2 and 5 years were 583 (95% CI 84–1074), 1527 (95% CI 310–2697) and 2804 (95% CI 695–4990), respectively.

### AGE-associated hospitalisations

**Fig. 4.**
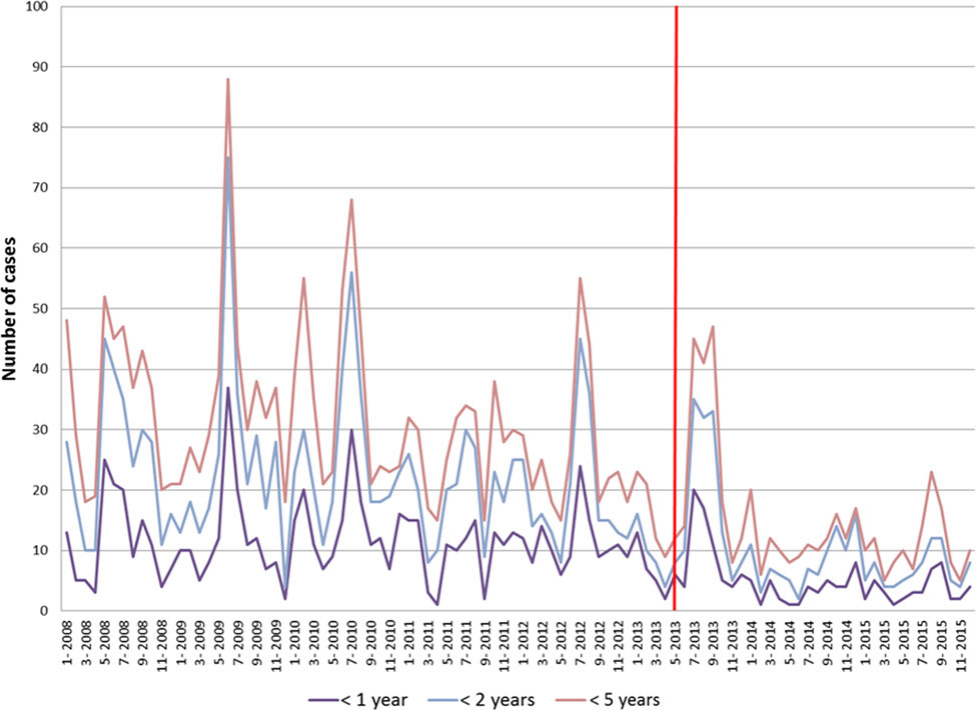
Number of AGE-associated HDs per calendar month for different age groups in San Luis.

**Fig. 5.**
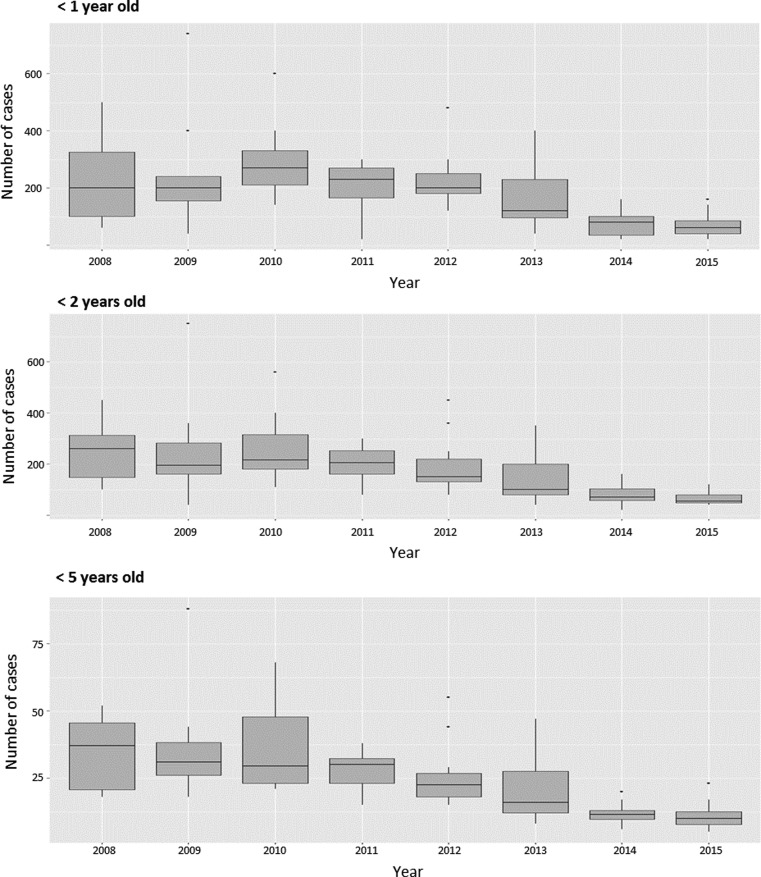
Boxplot of the distribution of the number of AGE-associated HDs per month each year of the study period in children in different age groups in San Luis. AGE, acute gastroenteritis.

Figure 4 presents the monthly number of AGE-associated HDs for the different age groups in San Luis from January 2008 to the end of 2015. A similar graph for Mendoza is presented in Figure SM4. In each graph, the vertical red line indicates the respective start dates of RV vaccination. The boxplot diagram in Figure 5 shows the distribution per year of the monthly number of AGE-associated hospitalisations in San Luis. A falling trend over the period from 2008 to 2012 is discernible in each age group.

#### Time-trend analysis for AGE-associated HD in San Luis (2008–2015)

The results summarised in the upper panel of Table 2 show a relative reduction in the number of AGE hospitalisations of children aged <5 years of 56% (95% CI 40%–74%) and slightly higher relative reductions for the two youngest groups. The cumulative number of AGE-associated HDs averted until the end of 2015 in children aged less than 1, 2 and 5 years were 237 (95% CI 146–339), 448 (95% CI 276–615) and 603 (95% CI 428–808), respectively.

**Table 2.** Time-trend analysis of AGE-associated HDs per month in San Luis with and without adjustment using Mendoza data as control

	Average number of HDs pre-vaccination	Average number of HDs post-vaccination	Predicted number of HDs without vaccination HD (95% CI)	Absolute effect averted # of HD (95% CI)	Relative effect observed (95% CI)	*P* Value
Age group						
Model 1: Time-trend analysis for San Luis (2008–2015)						
<1 year	12	5	12 (10–16)	−7 (−5 to −11)	−60% (−37% to −86%)	0.001
<2 years	22	10	24 (19–29)	−14 (−9 to −19)	−58% (−36% to −80%)	0.001
<5 years	32	15	34 (28–40)	−19 (−13 to −25)	−56% (−40% to −74%)	0.001
Model 2: Time-trend analysis for San Luis adjusted by Mendoza as control (2008–2014)						
<1 year	12	6	14 (11–17)	−8 (−5 to −11)	−59% (−37% to −80%)	0.001
<2 years	22	12	27 (21–33)	−15 (−9 to −21)	−55% (−34% to −77%)	0.001
<5 years	31	17	37 (31–43)	−20 (−14 to −26)	−54% (−38% to −72%)	0.001

HD, Hospital discharges; CI, confidence interval; #, Number; AGE, acute gastroenteritis.

Regarding the cumulative number of cases averted and the relative reduction in the 1 < 2 and 2 < 5 groups were 210 (95% CI 121–302) cases averted and 56% reduction (95% CI 32%–81%) for the 1 < 2 and 155 (95% CI 98–214) cases averted and 51% reduction (95% CI 32%–71%) respectively.

#### Time-trend analysis for AGE-associated HD in San Luis using Mendoza data as control (2008–2014)

With the prediction model adjusted by Mendoza data as a control, the average monthly number of AGE-associated hospitalisations for children aged <5 years was estimated to be reduced by 20 HDs or a statistically significant relative reduction of 54% (95% CI 38–72) with slightly higher relative reductions for the two youngest age groups (Table 2, lower part).

**Fig. 6.**
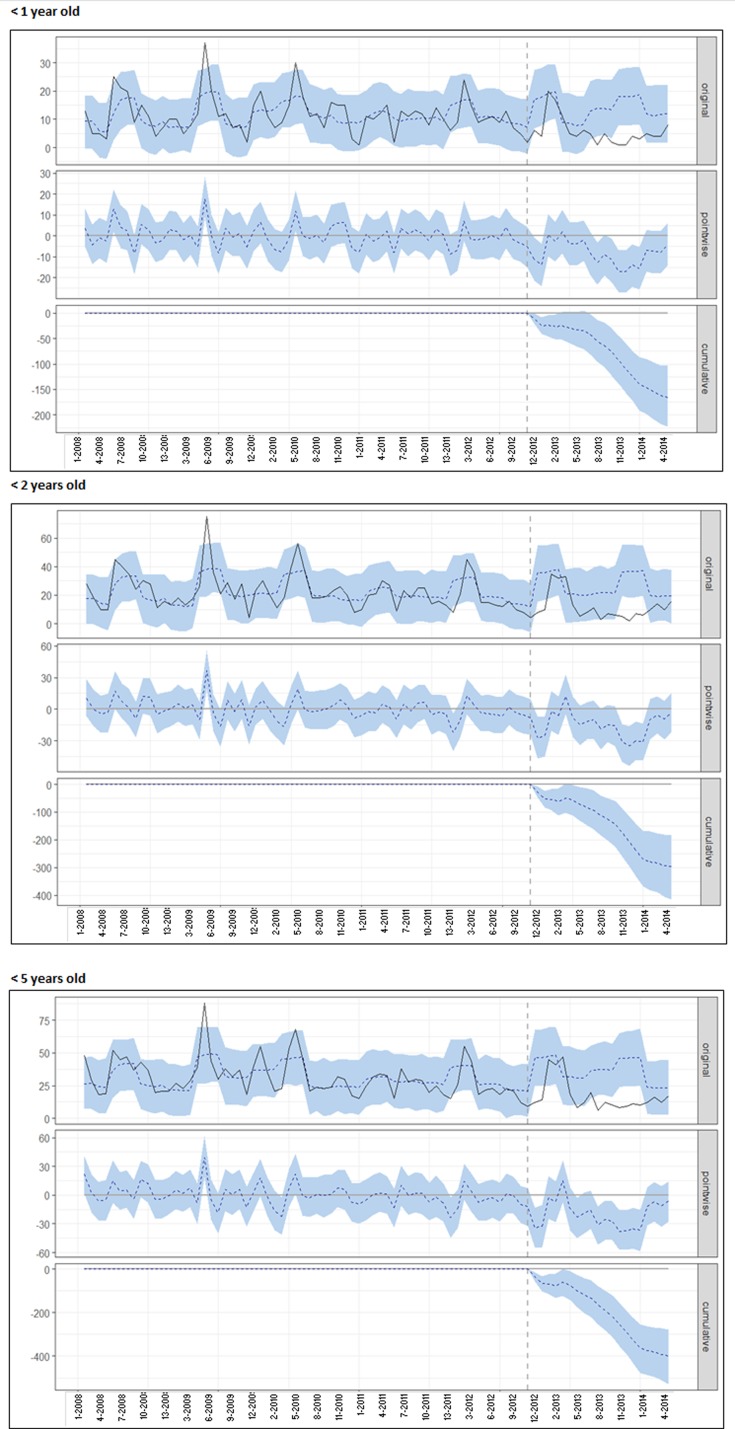
Time-trend analysis for AGE-associated HDs in San Luis for children in different age groups, after adjusting with Mendoza data as the control group. This figure shows the number of cases or HDs for the different age groups by epidemiological week (starting in January 2008). The upper graph (original) contains the observed values (full line) and the model predicted values in the case of no intervention (dotted line). The middle (pointwise) graph shows the difference between the observed and predicted value by period under analysis and the lower (cumulative) graph shows the cumulative difference throughout the period under analysis. The vertical dotted line represents the time of the introduction of the vaccine (May 2013). The areas shadowed in light blue refer to the 95% CI of the estimate in the upper graph (original), of the difference for each time point (pointwise) in the middle graph and of the cumulative difference in the lower graph (cumulative). AGE: acute gastroenteritis.

Figure 6 shows the time-trend analysis for AGE-related HD for San Luis (until the end of 2014), using Mendoza data as control. For each age group, the top panel shows the observed (full line) and predicted (dotted line) number of AGE-associated HDs; the middle panel shows for each time point the difference between the number of observed and predicted AGE-related HDs and the bottom panel shows the cumulative number of AGE-associated HDs averted from vaccine introduction until the end of 2014. For children aged less than 1, 2 and 5 years, the cumulative number of AGE-associated HDs averted were 165 (95% CI 103–223), 297 (95% CI 184–415) and 399 (95% CI 277–528), respectively.

Regarding the cumulative number of cases averted and the relative reduction in the 1 < 2 and 2 < 5 groups were 132 (95% CI 76–189) cases averted and 51% reduction (95% CI 29%–74%) for the 1 < 2 and 107 (95% CI 71–145) cases averted and 52% reduction (95% CI 34%–70%) respectively.

### Direct healthcare costs avoided

Assuming direct healthcare costs of 155 Argentine pesos per AGE case and 10 340 Argentine pesos per AGE-associated hospitalisation, the estimated cumulative 2804 AGE cases and 399 AGE-associated hospitalisations averted in San Luis from May 2013 until the end of 2014 would have led to a reduction of direct healthcare costs of 4 560 280 Argentine pesos (corresponding to US$ 524 170 using 2014 exchange rate from the World Bank).

## Discussion

**Fig. 7.**
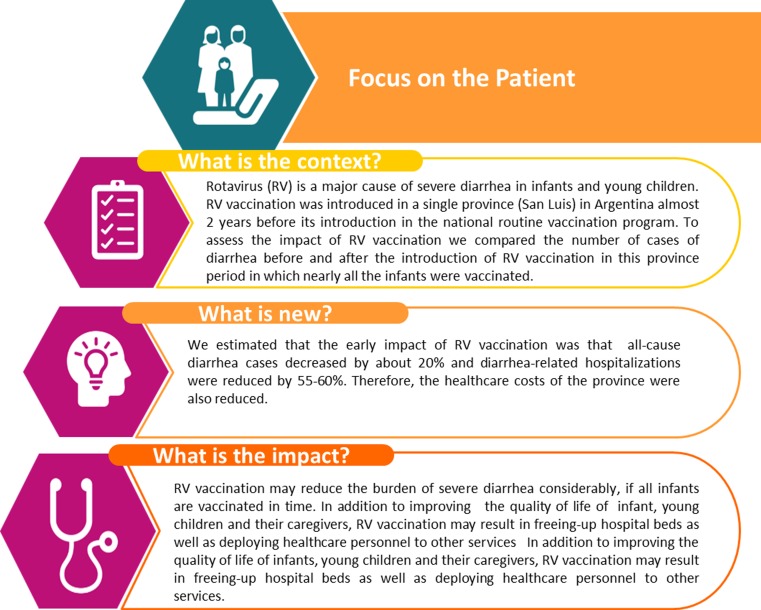
Lay and plain language message regarding the context and the impact of the intervention.

The observed results in San Luis province regarding the impact of RV vaccination with and without adjustment by data from Mendoza resulted in quite similar estimates. Figure 7 presents a summary of the outcomes and the impact of this study for healthcare providers. The impacts observed were relative reductions of ~20% in the number of all-cause AGE cases and of 55–60% in the number of AGE hospitalisations. There was a tendency that the impact was a little greater for infants aged <1 year than for the older age groups. The associated reduction in direct medical costs of outpatient care and hospitalisations amounted to more than 4.5 million Argentine pesos in San Luis over the period from May 2013 to the end of 2014.

Previous ecological studies of the impact of RV vaccination in Latin American countries have generally focused on all-cause AGE-related mortality and hospitalisations because data on laboratory-confirmed RV-positive cases are normally not available at the population level. Early studies from four countries (Brazil, El Salvador, Mexico and Panama) reported reductions of all-cause AGE hospitalisations ranging between 17% and 51% in children aged <5 years [14–18]. A few studies with RV-specific data available reported reductions of RVGE hospitalisations ranging from 59% to 81% in <5-year-olds [19, 20]. Large reductions in mortality of severe diarrhea have also been reported [14, 15, 21].

Newer studies tend to report more limited impact. Combining data from four countries (Bolivia, El Salvador, Honduras and Venezuela), De Oliveira *et al*., reported reductions in all-cause AGE hospitalisations of 12.4% in children aged <1 year and 8.5% in children aged <5 years. The range across countries was 7.5–27.0% for the 0- to 1-year-olds and 5.6–17.9% for the 0- to 5-year-olds [22]. A renewed analysis in Brazil with data for at least 5 years after RV vaccine introduction showed a sustained reduction in all-cause AGE hospitalisations of 31% in children aged <1 year and 22.4% in children aged 1–4 years [23].

Although the point estimates of the reduction in all-cause AGE hospitalisations vary widely between these disease-burden trend analyses, they are consistent in estimating a statistically significant impact. Our results are in line with this but with larger estimated reductions in all-cause AGE hospitalisations than in the previous studies. No other study of the impact of RV vaccination on the overall number of all-cause AGE cases in Latin America has been identified.

RVGE in infants is often more severe than AGE of other causes. Thus, it has been estimated that the proportions of RVGE cases among all-cause AGE cases are 5–10% for cases taken care of by the child's parents or caretakers in the home, 15–20% of cases presenting in primary care or outpatient settings and 30–50% of hospitalised cases [24]. The proportions found in Argentina correspond to this, with RVGE accounting for 17% of all AGE cases [7] and 42% of hospitalised AGE cases [8]. That the estimated relative reduction in AGE outcomes is approximately three times higher for all-cause AGE-related hospitalisations than for the overall number of AGE cases presenting for medical care is therefore suggestive of an effect of RV vaccination.

Our assumption that the reduction in all-cause AGE cases and hospitalisations could be attributed to RV vaccination is supported by the lack of evidence on any changes in the registration of AGE cases during the study period. Its robustness was furthermore tested by randomly selecting five hypothetical time points for the introduction of RV vaccination in San Luis and testing the statistical prediction models based on these sham intervention time points. These tests showed no significant deviation between the predicted and observed outcomes and this was taken to support the robustness of the assumption and the model. Previous RV-vaccination impact studies generally make similar assumptions.

In a recent systematic review and meta-analysis of case-control studies of the effectiveness of RV vaccination in Latin America, Santos *et al*. found that RVGE amounted to 24% and 16% of all AGE cases before and after the introduction of RV vaccination, respectively. Their estimates of vaccination effectiveness were 53% against RV infection, 74% against severe RVGE and 73% against RVGE hospitalisations [5]. It seems reasonable to expect that these RVGE-specific estimates of the effectiveness of RV vaccination should be broadly applicable to Argentina also.

A self-evident limitation of our study is that all-cause AGE was used as the outcome, so it is not possible to obtain a RVGE-specific estimate of the impact of the RV vaccine. However, it may arguably be the case that estimating the impact on all-cause AGE is more valuable for decision making in providing an estimate of the fraction of AGE outpatient cases and hospitalisations preventable by RV vaccination.

Even if RVGE cases were identified, the ecological study design precludes definitive conclusions about the impact of RV vaccination, because such studies do not link an outcome to an exposure at the individual level. Herd effects with indirect protection of unvaccinated children complicate the assessment of a causal association between vaccination coverage and disease patterns. That we found almost the same relative reduction for the age group <5 years as for the two youngest groups may suggest that herd protection occurred because a large proportion of the children in the <5 years group were too old to be vaccinated in the setting of the provincial vaccination program. Herd effects have been observed in many studies after the introduction of general RV vaccination with a range of effects from 0 to 72%, varying considerably between consecutive years; generally, the estimated effect diminishes with increasing age of the children (studies cited in [25]).

With only 1.5 years of observation post vaccination this impact study is provisional and to assess the medium- to long-term impact of RV vaccination with possible serotype replacement or changes in seasonality or the disease profile, continued surveillance at the national level is required. The almost complete coverage obtained in San Luis may be a cause of the high impact, the 2-dose coverage observed in Argentina after the introduction of RV vaccination in the national vaccination program in January 2015 is much lower (85% in 2015 and 74% in 2016).

## Conclusions

Similar to the findings of many other studies of the early impact of RV vaccination we found a higher effect of RV vaccination in preventing more severe AGE cases requiring hospitalisation than in preventing all cases of AGE presenting for medical care. We also showed that RV vaccination led to substantial immediate savings of direct medical costs in the San Luis province.

## Supplementary material

For supplementary material accompanying this paper visit https://doi.org/10.1017/S0950268819001936. click here to view supplementary material
