# Impact assessment of the incorporation of the rotavirus vaccine in the province of San Luis – Argentina- CORRIGENDUM

**DOI:** 10.1017/S0950268819002164

**Published:** 2019-12-12

**Authors:** S. García Martí, F. Augustovski, L. Gibbons, V. Loggia, A. Lepetic, J.A. Gómez, A. Pichón Riviere

The above paper was originally submitted to *Epidemiology & Infection* with the indication that A. Pichón Riviere's surname should be indexed as ‘Riviere, AP’ instead of ‘Pichón Riviere, A.’ and was originally published as such.

This has been rectified in the online PDF and HTML copies and re-indexed accordingly.
