# Supplementary material for: Impact assessment of the incorporation of the rotavirus vaccine in the province of San Luis – Argentina
Source: Epidemiol Infect. 2019 Nov 27;147:e308. doi: 10.1017/S0950268819001936 (PMC7003627; doi:10.1017/S0950268819001936)

**Supplementary Material**

**Figure SM1. Number of AGE cases for different age groups per 4-week periods for Mendoza.**


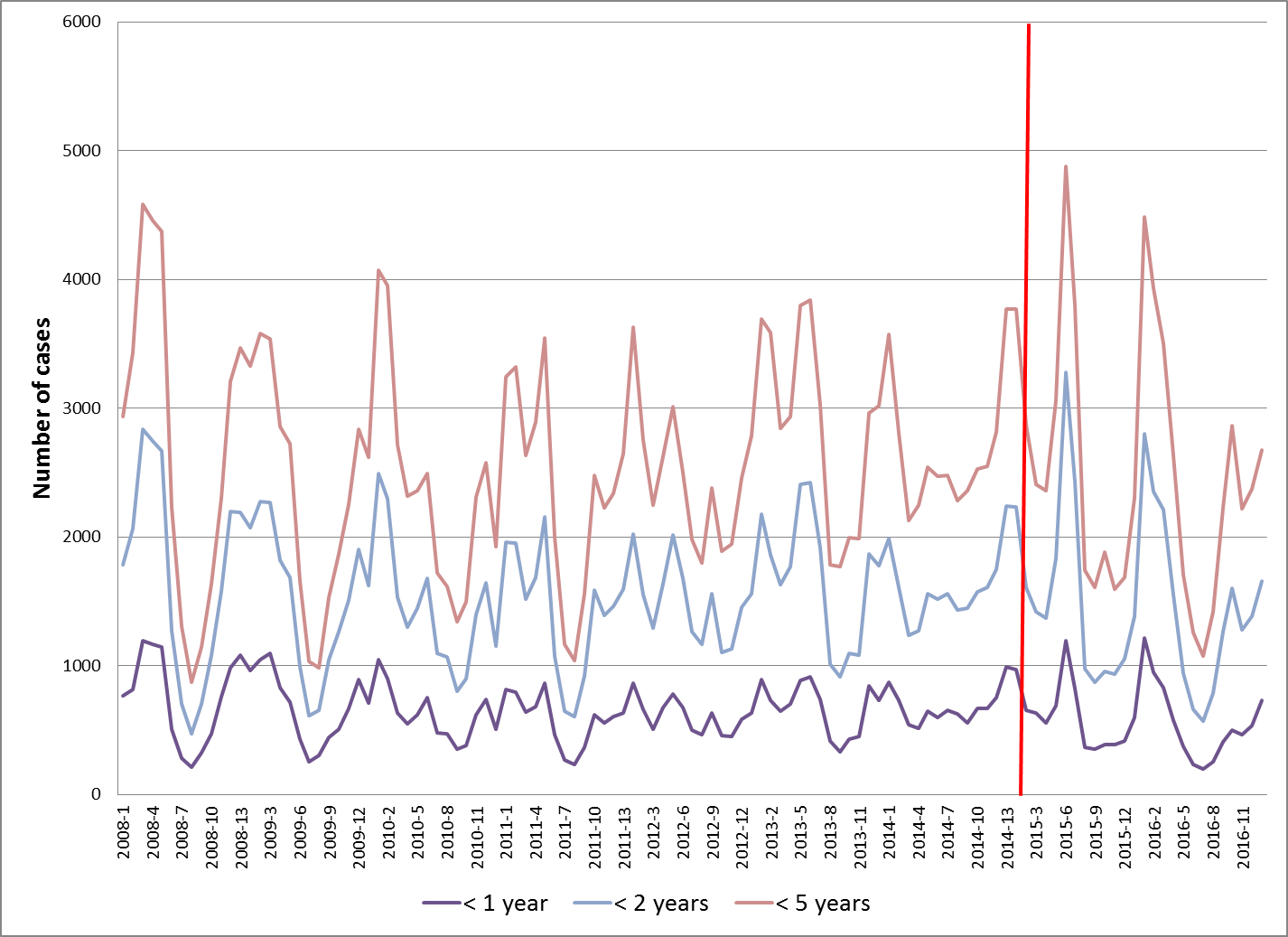


**Figure SM2. Time trend analysis for AGE cases in different age groups in San Luis, without taking into account adjusting for Mendoza data as control group**

A. Less than 1 year old


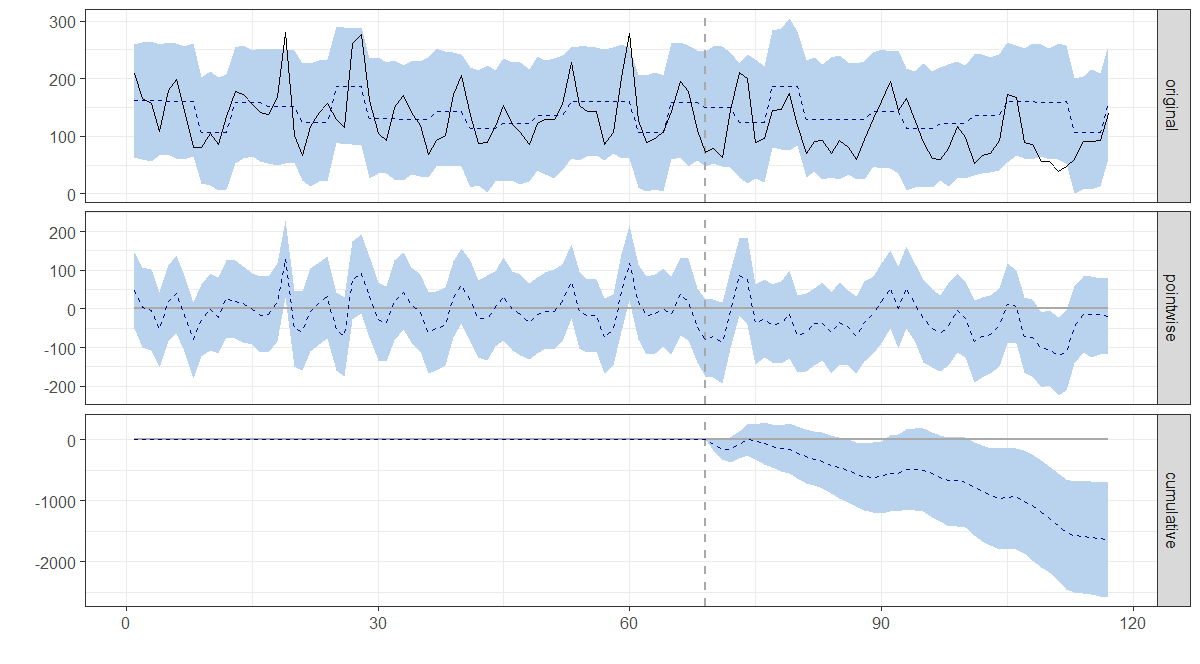


B. Less than 2 year old


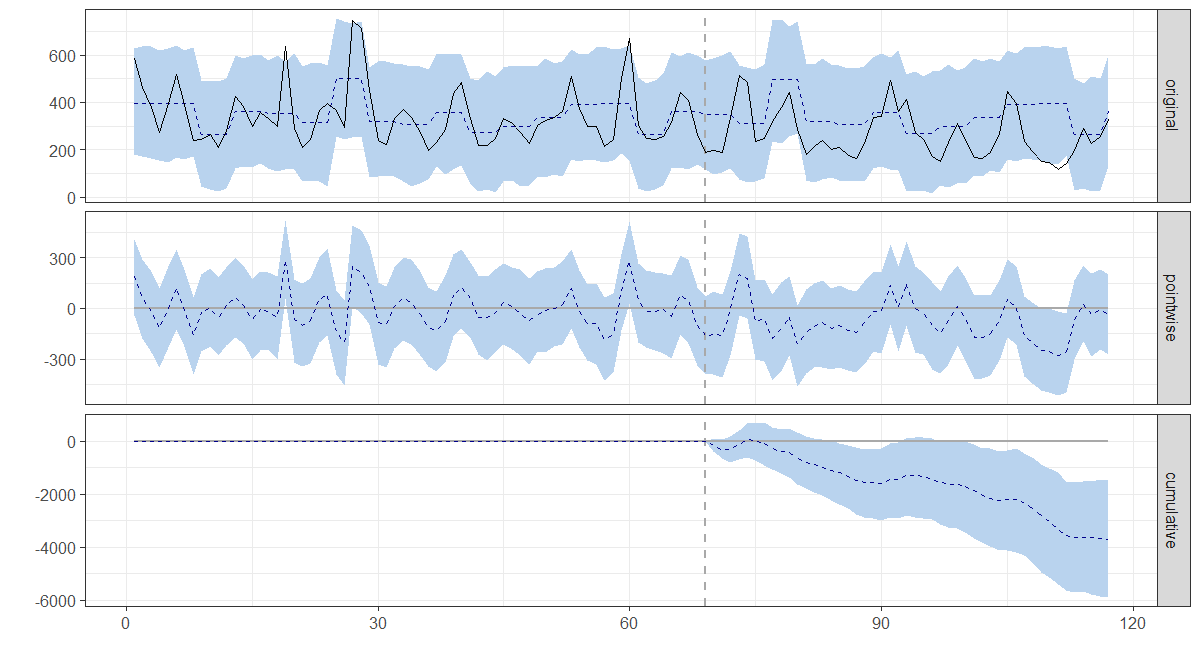


C. Less than 5 year old


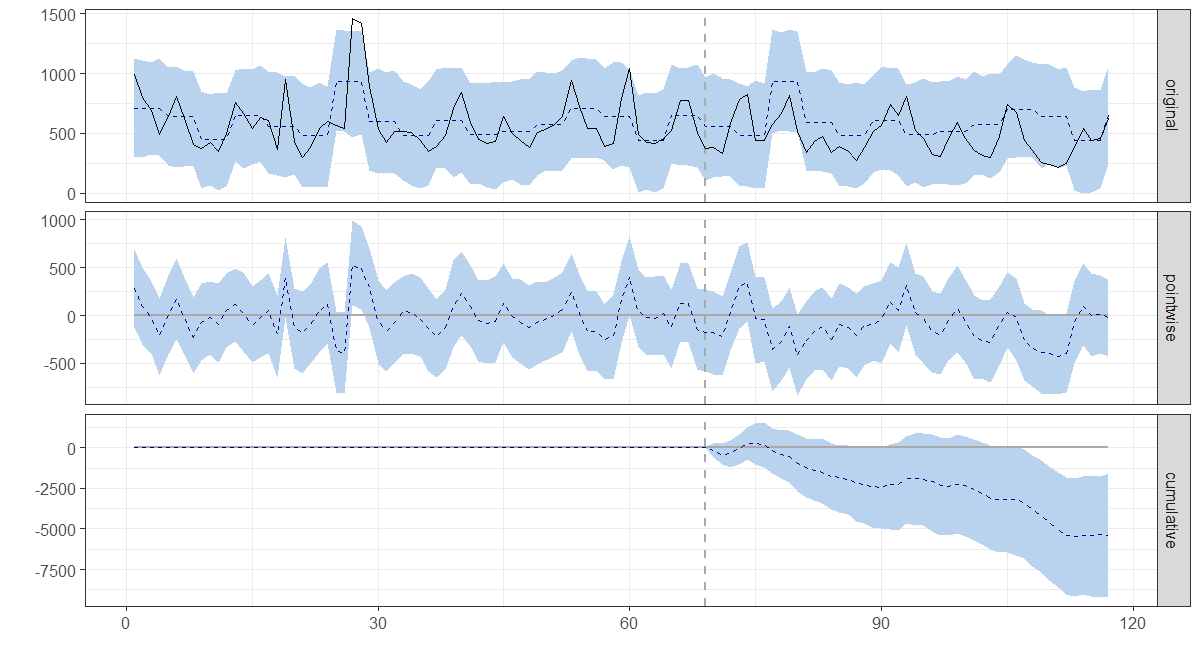


Figure SM2 shows number of cases or hospital discharges for the different age groups by epidemiological week (starting in January 2008). The upper graph (original), contains the observed values (full line) and the model predicted values in case of no intervention (dotted line). The middle (pointwise) graph show the difference between observed and predicted value by period under analysis and lower (cumulative) graph shows the cumulative difference throughout the period under analysis. The vertical dotted line represents the time of introduction of the vaccine (May 2013). The areas shadowed in light blue refer to the 95% confidence intervals of the estimate in the upper graph (original), of the difference for each time point (pointwise) in the middle graph and of the cumulative difference in the lower graph (cumulative). AGE: acute gastroenteritis

**Figure SM3. Time trend analysis for AGE-associated hospital discharges in San Luis for children in different age groups, without taking into account adjusting for Mendoza data as control group**

A. Less than 1 year old


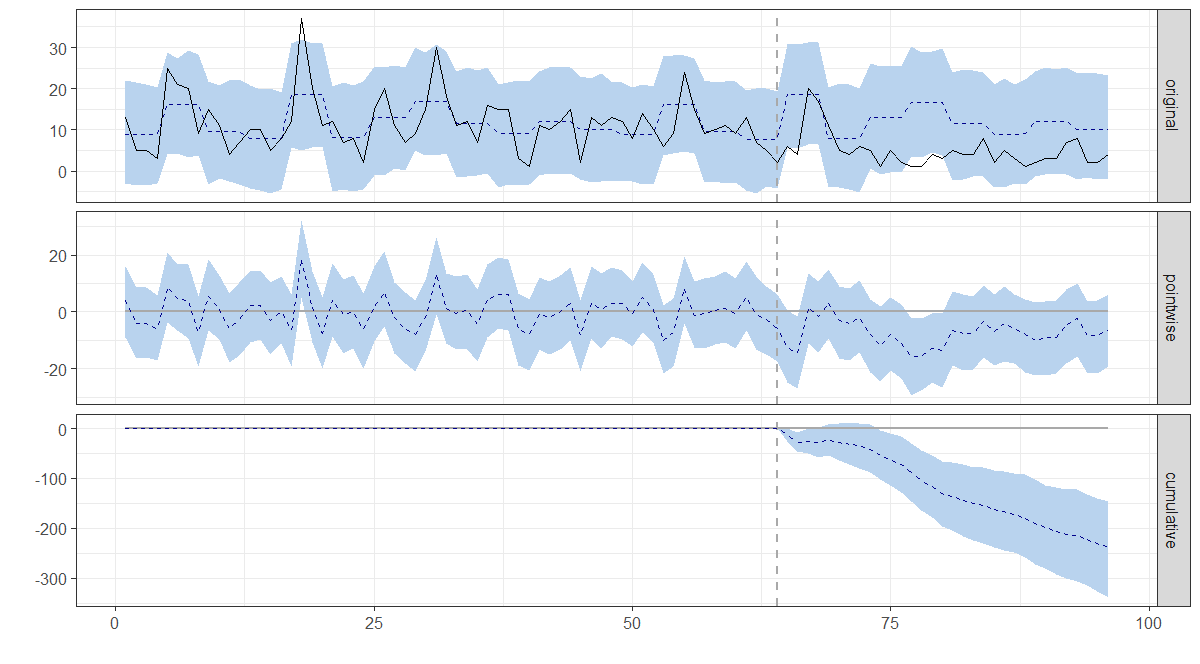


B. Less than 2 year old


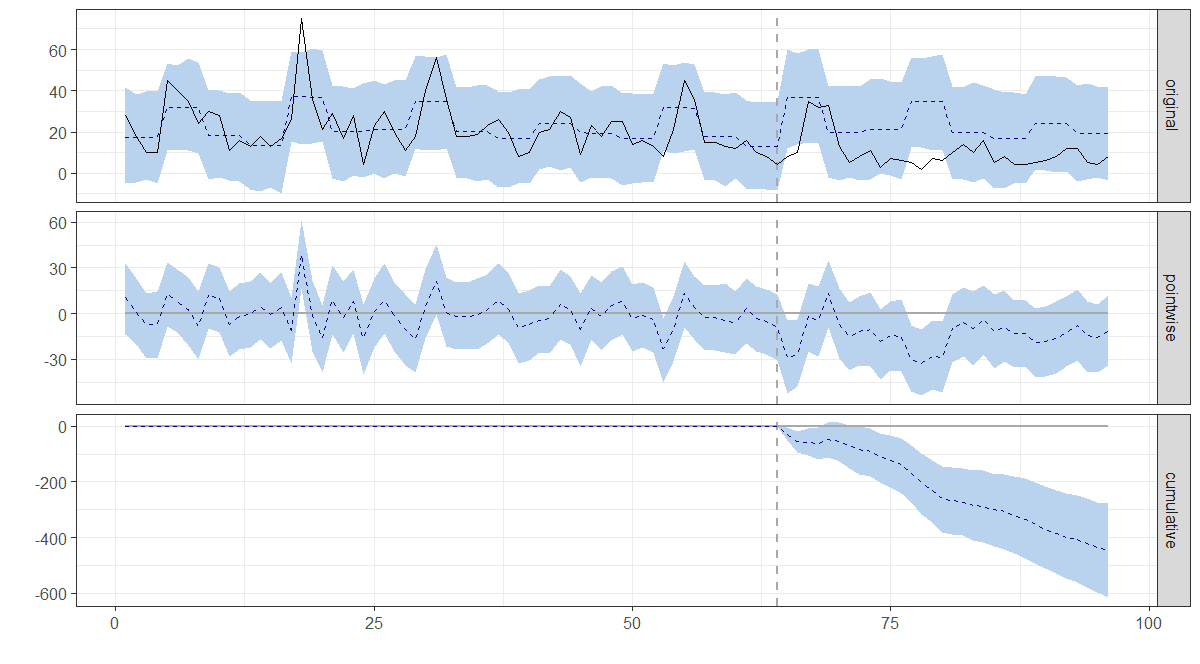


C. Less than 5 year old


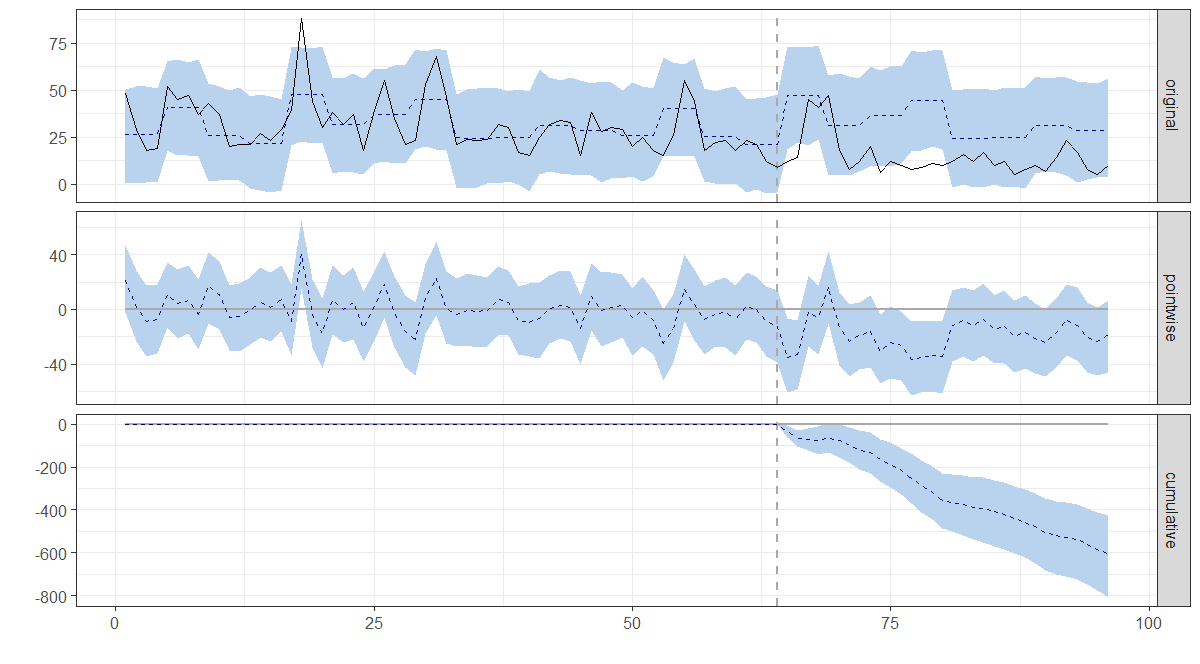


Figure SM3 shows number of cases or hospital discharges for the different age groups by epidemiological week (starting in January 2008). The upper graph (original), contains the observed values (full line) and the model predicted values in case of no intervention (dotted line). The middle (pointwise) graph show the difference between observed and predicted value by period under analysis and lower (cumulative) graph shows the cumulative difference throughout the period under analysis. The vertical dotted line represents the time of introduction of the vaccine (May 2013). The areas shadowed in light blue refer to the 95% confidence intervals of the estimate in the upper graph (original), of the difference for each time point (pointwise) in the middle graph and of the cumulative difference in the lower graph (cumulative). AGE: acute gastroenteritis

Figure SM4. Number of AGE-associated hospital discharges per calendar month for different age groups in Mendoza.


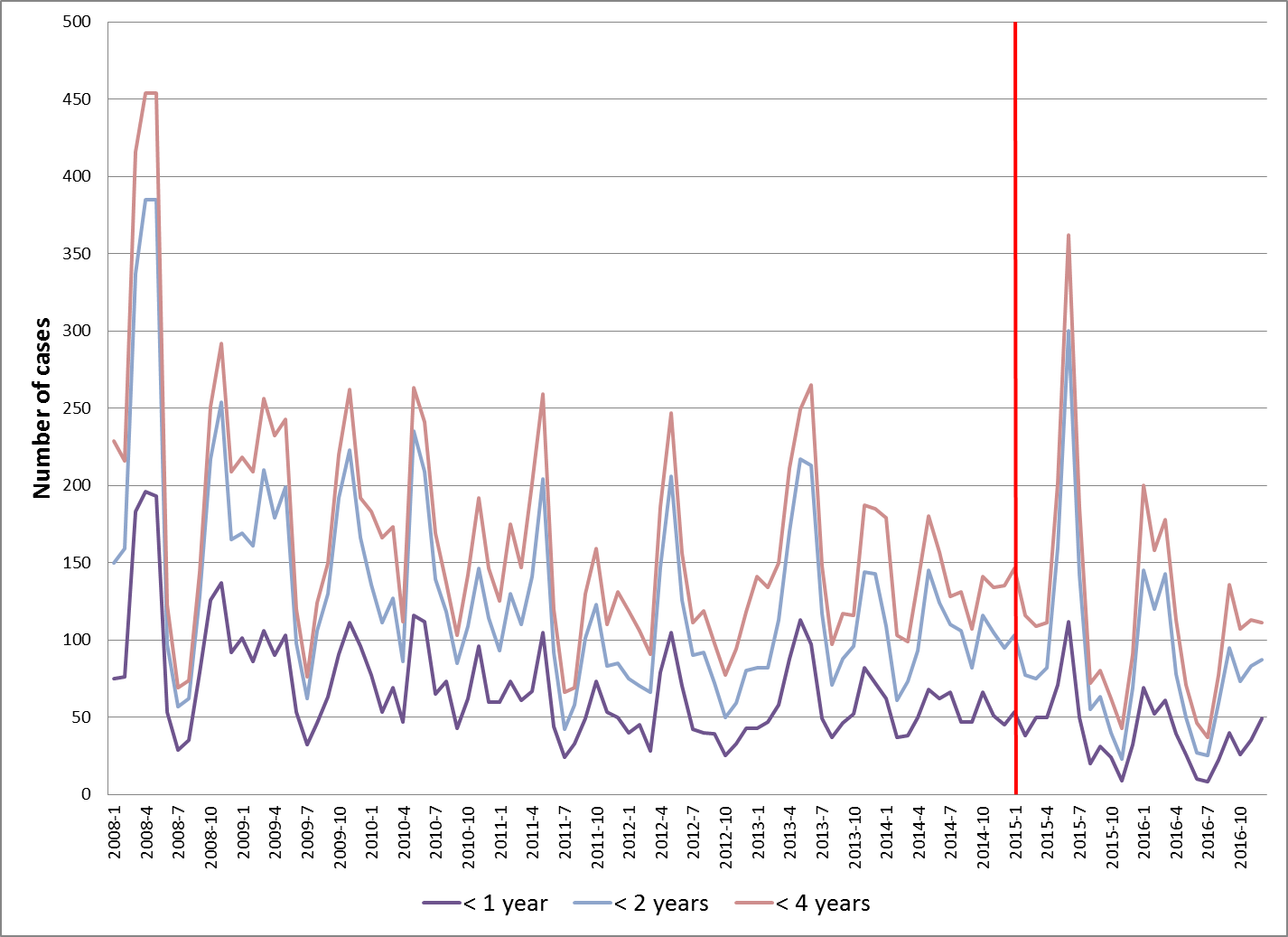

Supplement: Supplementary file 1 [file S0950268819001936sup.zip › S0950268819001936sup002.docx]
